# Personal

**Published:** 1906-12

**Authors:** 


### PERSONAL.

---

DR. J. FORD THOMPSON, of Washington, who went abroad for the benefit of his health sometime ago, has been seriously ill in London and recently submitted to a grave surgical procedure, the nature of which is not known to us at this writing, but which has resulted favorably, according to late cable dispatches. Dr.

Thompson has been in practice about fifty years and for the greater portion of that time has enjoyed the reputation of being the leading surgeon in the capital,—indeed, no one has excelled him in this respect south of the boundary established by Mason and Dixon. Dr. Thompson was bereaved of his wife a year or two ago, which was a great shock to him. Mrs. Thompson was an accomplished woman and a leader in every walk where woman's better influence is needed.

We tender to Dr. Thompson our best wishes for a speedy and complete recovery.

---

Dr. L. S. McMurtry, of Louisville, read a paper on 'Tuberculosis of the Peritoneum" before the Cleveland Academy of Medicine in the new home of the profession in that city Friday evening, November 16, 1906. The medical profession of Cleveland occupies a handsome building equipped with a voluminous library, reading rooms, auditorium, and other conveniences. This makes the Buffalo medical accomodations, by contrast, look poor indeed.

---

Dr. John H. Pryor, of Buffalo, has been appointed a member of the board of trustees of the New York State Hospital for the treatment of incipient tuberculosis at Raybrook in the Adirondacks, vice Dr. Willis G. Macdonald of Albany, resigned. Dr. Prior was a pioneer in working for the establishment of this hospital and was appointed a trustee when the board was first created. He was immediately promoted to the office of superintendent, which he filled for about two years. He worked indefatigably to put the hospital on a high professional plane, but resigned because he was unable to carry out his ideals, owing to interferences from Albany. Dr. Pryor's return to the board is a good omen and we believe will result in progressive and efficient management of the affairs of the hospital. He will, of course, continue his professional practice in Buffalo, making periodic visits to the hospital to attend the meetings of the board.

---

Dr. L. W. Bremerman, of New York, has been apointed professor of Genitourinary Diseases in the New York School of Clinical Medicine, to fill the vacancy caused by the death of Dr. William K. Otis.

---

Dr. Roland O. Meisenbach, of Buffalo, has removed his residence to 122 Bidwell Parkway. His office remains at 140 Allen street. Hours, 1 to 3. Telephone, Tupper 104.

Dr. James H. Lewis, of Buffalo, has removed his office from 90 North Pearl Street, to 520 Franklin Street, between Allen and North. Hours, 1 to 3; 7 to 8. Telephone, Tupper 61.

---

Dr. Ernest Wende, who was appointed sometime ago to be Commissioner of Health of Buffalo, will take office January 1, 1907. Dr. Wende held the office from 1892 to 1902, and is well qualified to take up its duties again.

---

Dr. Francis E. Fronczak has been appointed assistant health commissioner of Buffalo, and will enter upon the duties of the office January 1, 1907.

---

Dr. Jacob Goldberg, of Buffalo, has been appointed School Examiner, vice Dr. P. W. Van Peyma who declines reappointment. Dr. Goldberg's duties will commence at the beginning of the New Year.

---

Dr. William Gaertner, of Buffalo, has been appointed a trustee of the Grosvenor Public Library, vice Josiah Jewett, whose term expires March 11, 1907.

---

Dr. D. P. Card, of Utica, formerly of the Bellevue Hospital staff, New York, has been appointed assistant surgeon of the Soldiers and Sailors Home, at Bath, N. Y., to fill a vacancy caused by the resignation of Dr. Spencer L. Higgins.

